# Diagnosing malignant distal bile duct obstruction using artificial intelligence based on clinical biomarkers

**DOI:** 10.1038/s41598-023-28058-5

**Published:** 2023-02-24

**Authors:** Yuichi Sugimoto, Yusuke Kurita, Takamichi Kuwahara, Motokazu Satou, Koki Meguro, Kunihiro Hosono, Kensuke Kubota, Kazuo Hara, Atsushi Nakajima

**Affiliations:** 1 Department of Gastroenterology, Yokohama Sakae Kyosai Hospital, Yokohama, Japan; 2 Department of Gastroenterology and Hepatology, Yokohama City University School of Medicine, Yokohama, Japan; 3 Department of Gastroenterology, Aichi Cancer Center Hospital, Nagoya, Japan

**Keywords:** Pancreatic disease, Biliary tract disease

## Abstract

Diagnosing distal bile duct obstruction remains challenging. This study aimed to examine the diagnostic ability of artificial intelligence (AI) based on clinical biomarkers in diagnosing malignant distal bile duct obstruction. A total of 206 patients with distal bile duct obstruction were included in this study. Clinical laboratory parameters were collected from the patients and evaluated using AI. All clinical parameters were input into the AI algorithm, and the AI value for malignant distal bile duct obstruction was calculated. The benign and malignant diagnostic capabilities of AI and other factors (alkaline phosphatase [ALP], intrahepatic bile duct [IHBD] diameters, and total bile duct [CBD] diameters) were compared. Benign and malignant bile duct obstruction were diagnosed in 142 and 64 patients, respectively. The median AI value of malignant distal bile duct obstruction was significantly greater than that of benign distal bile duct obstruction (0.991 vs. 0.002, *p* < 0.001). The area under the receiver operating characteristic curve of AI, ALP, IHBD diameter, and CBD diameter were 0.908, 0.795, 0.794, and 0.775, respectively. AI showed a sensitivity, specificity, and accuracy of 83.1%, 87.2%, and 85.9%. AI-based on clinical biomarkers could serve as an auxiliary for diagnosing malignant bile duct obstruction.

## Introduction

Distal bile duct obstruction may occur not only in benign diseases, such as choledocholithiasis, chronic pancreatitis, and immunoglobulin G4 (IgG4)-related sclerosing cholangitis, but also in malignant diseases, such as distal bile duct and pancreatic cancers^1^. Distal bile duct obstructions, especially malignant ones, often require surgical resection and chemotherapy. Therefore, an accurate diagnosis of malignant bile duct obstruction is necessary.

Transabdominal ultrasonography (US), computed tomography (CT), magnetic resonance cholangiopancreatography (MRCP), and endoscopic examinations, including endoscopic retrograde cholangiopancreatography (ERCP), are performed to diagnose distal bile duct obstruction. In lower bile duct obstructions, the diagnostic accuracies of US, multi-detector row CT, and plain MRCP for malignant lesions are 36.8%^2^, 73.0%^3^, and 78.0%^4^, respectively. As a result, accurately diagnosing and differentiating benign from malignant duct obstruction using imaging modalities are still challenging. Although a pathological diagnosis is necessary for a definitive diagnosis, the sensitivity for pathological malignancy by cytology with ERCP and endoscopic transpapillary forceps biopsies in patients with distal bile duct obstruction is 30–48%^5^ and 59%^3^, respectively. Hence, the accuracy of pathological diagnosis is inadequate. It has also been reported that 15–24% of cases in which biliary tract cancer was suspected were benign^6^. Therefore, preoperative differentiation of benign and malignant distal bile duct obstruction remains challenging.

In recent years, artificial intelligence (AI) advancements have been remarkable^7,8^. AI has been used in biliary-pancreatic diseases, relatively common in intrahepatic cholangiocarcinoma, and a wide variety of modalities have been reported, including CT, magnetic resonance imaging (MRI)^9^, and various AI models such as artificial neural network (ANN)^10^ and support vector machine (SVM)^11^. In the case of extrahepatic cholangiocarcinoma, the radiomics models of MRI through random forests showed an area under the curve (AUC) of 0.8 and 0.9 for the detection of differentiation degree and lymph node metastasis^12^, respectively, but there are no reports on diagnoses of benign and malignant distal bile duct obstruction. As it is known, the diagnosis or differential diagnosis of distal bile duct obstruction is sometimes difficult. It requires much experience and knowledge because of the variety of radiological features of each disease and the overlap of radiological findings between many diseases. Therefore, in this study, we examined the diagnostic ability of AI using clinical laboratory parameters in differentiating malignant from benign distal bile duct obstruction.

## Method

### Patients and feature selection

We retrospectively reviewed all patients whose distal bile duct obstruction or dilated upstream bile duct was detected on CT scan and underwent ERCP from April 2015 to August 2020 at Yokohama Sakae Kyosai Hospital. Bile duct abnormalities were considered to be involved when the radiologists encountered: (1) bile duct dilatation (7 mm for the common bile duct [CBD]), or (2) biliary wall thickening (1 mm), or (3) masses inside or around the affected duct. Diseases of benign bile duct obstruction included choledocholithiasis, IgG4-related sclerosing cholangitis, chronic pancreatitis, and other benign stenoses. Malignant bile duct obstruction diseases included distal bile duct cancer, pancreatic cancer, ampullary cancer, lymphoma, recurrent lymph node of gastric carcinoma, and duodenal carcinoma. The clinical laboratory parameters of the patients that were evaluated in this study were extracted before ERCP. All laboratory biomarkers were first examined before biliary drainage. The parameters were age, sex, weight, height, intrahepatic bile duct (IHBD) diameter, CBD diameter, leukocyte count (WBC), and WBC fraction (neutrophils and lymphocytes). The values of total bilirubin (T-bil), aspartate aminotransferase (AST), alanine aminotransferase (ALT), γ-glutamyl transpeptidase (γ-GTP), alkaline phosphatase (ALP), amylase, albumin, lactate dehydrogenase (LDH), C-reactive protein (CRP), hemoglobin A1c (HbA1c), glucose, triglyceride (TG), low-density lipoprotein (LDL), high-density lipoprotein (HDL), total cholesterol (T-chol), carcinoembryonic antigen (CEA), carbohydrate antigen 19–9 (CA19-9), and concurrent biliary infection were also assessed. Parameters that had more than half of the missing data were excluded beforehand. Extraction of the clinical information and AI analysis was done by different authors, blind to each other.

This study was approved by the Institutional Review Boards of Yokohama Sakae Kyosai Hospital (20,201,019-2), Aichi Cancer Center Hospital (2021-1-044) and performed in accordance with the Declaration of Helsinki^13^. This retrospective observational study used only medical information and did not compromise the privacy of the participants. All patients received an opt-out form for informed consent. Those who did not agree to participate were excluded. All authors had access to the research data and reviewed and approved the final manuscript.

### Definition of final diagnosis

Histopathological examination of the surgical specimens was considered the final diagnosis in cases of surgical resection. The final diagnosis of patients who did not undergo surgery was determined by either biopsy, cytology, clinical data, or imaging with a 1-year follow-up.

Regarding the diagnosis of malignancy, surgical specimens were used in surgical cases, and biopsy specimens or cytology were used in non-surgical cases. The benign disease was diagnosed if there were no malignant findings on surgical specimens, biopsy specimens, or cytology, and there was no change in the imaging results after at least a 1-year follow-up period. The diagnosis of choledocholithiasis was made following Tokyo Guidelines 2018^14^. The diagnosis of IgG4-related sclerosing cholangitis was made based on clinical diagnostic criteria of IgG4-related sclerosing cholangitis 2012^15^. Chronic pancreatitis was diagnosed per evidence-based clinical practice guidelines for chronic pancreatitis 2015^16^.

### AI algorithm

All machine learning methods used scikit-learn^17^. For the imputation of missing values, we used the K nearest neighbor algorithms. When predicting a certain value, we used K neighboring values (5 in this case) as a guide^18^. The synthetic minority oversampling technique^19^ and the undersampling method^20^ were used to correct the ratio of benign and malignant tumors. The Boruta method^21^ was used for feature selection.

For the machine learning method, we used light GBM (Microsoft.com)^22^, which is an easy-to-use (short training time) machine learning model used for the AI predictive model. Light GBM is based on gradient boosting^23^, which is an ensemble learning method using decision trees as weak learners^24^. The leaf-wise method^25^ was chosen to handle the decision tree. The predictive value of malignant probability using AI was continuous variables from 0 to 1. When the AI value was close to 1, the malignant probability increased. The tenfold cross-validation^26^ was used to accurately verify the generalization performance of the predictive model.

### Endpoint

The primary endpoint was to investigate and compare the diagnostic ability of AI, ALP, IHBD diameter, and CBD diameter using clinical laboratory parameters in differentiating malignant from benign lesions in patients with distal bile duct obstruction. We also investigated the diagnostic sensitivity, specificity, positive predictive value (PPV), negative predictive value (NPV), and accuracy of AI.

### Statistical analysis

Statistical analysis was performed using R software (version 4.0.2). Univariate analysis was performed using the Mann–Whitney U test for continuous variables. The Chi-square test or Fisher’s exact test was used for categorical variables. Statistical significance was set at *p* < 0.05. Receiver operating characteristic (ROC) curve analysis was performed to characterize the diagnostic ability of ALP, IHBD diameter, CBD diameter, and AI in differentiating malignant from benign distal bile duct obstruction. The area under the ROC curve (AUROC) was used to evaluate the accuracy of the quantitative tests.

## Results

**Figure 1:**
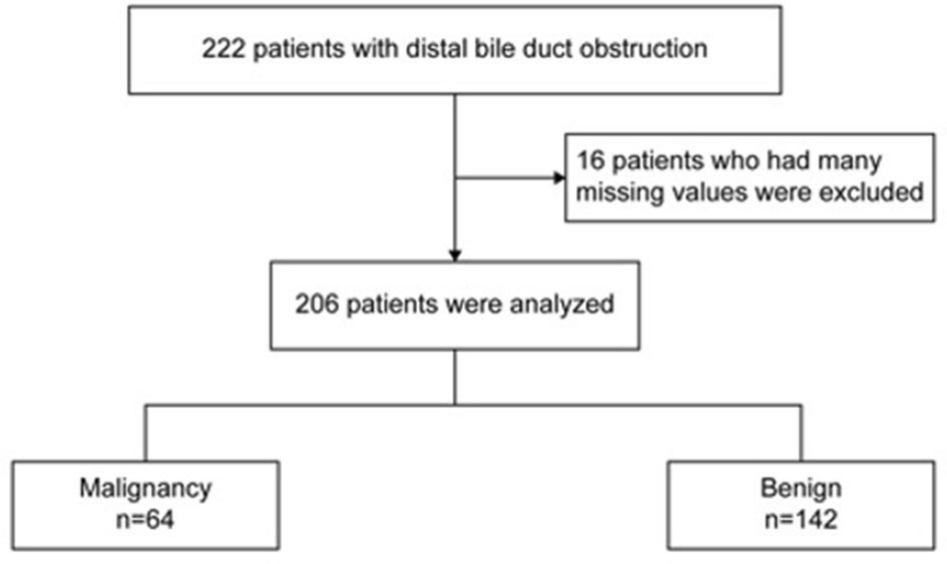
Flowchart of this study.

A total of 222 patients were identified; however, 16 were excluded due to missing data. Hence, 206 patients were included in the final analysis (Fig. 1). Benign and malignant bile duct obstructions were diagnosed in 142 and 64 patients, respectively. HbA1c, TG, LDL, HDL, T-chol, CEA, and CA19-9 were excluded beforehand, as these parameters had more than half the missing data. Characteristics of the patients are shown in Table 1. Factors used for the final AI analysis were IHBD diameter, CBD diameter, WBC, WBC fraction, T-bil, AST, ALT, γ-GTP, ALP, amylase, LDH, and CRP levels. The median IHBD diameter, CBD diameter, WBC count, ALP, ALT, γ-GTP, and T-bil of patients with malignant distal bile duct obstruction were significantly higher than those with benign distal bile duct obstruction. However, AST, LDH, amylase, and CRP levels were not significantly different between patients with malignant and benign distal bile duct obstruction.

**Table 1:** Baseline characteristics.

Variable	N = 206	Benign	Malignant	*p*
Patient		N = 142	N = 64	
Age (years), mean ± SD	78.2 ± 10.7	78.7 ± 11.1	77.2 ± 9.6	0.356
Sex (%)				0.453
Male	114 (55.3)	76 (53.5)	38 (59.4)	
Female	92 (44.7)	66 (46.5)	26 (40.6)	
IHBD diameter (mm), median (IQR)	5.4 (0–24.0)	3.0 (0–19.0)	10.0 (0–24.0)	< 0.001
CBD diameter (mm), median (IQR)	13.1 (4.0–30.0)	10.0 (4.0–27.0)	16.0 (4.0–30.0)	< 0.001
WBC (10^3^/μL)	8505 (1000–27,500)	8450 (1000–27,500)	6600 (3000–15,200)	< 0.001
Neutrophils (%)	75.9 (44.9–97.3)	80.5 (47.2–97.3)	70.8 (44.9–94.6)	< 0.001
Lymphocytes (%)	14.4 (0.3–46.2)	10.8 (1.3–37.3)	16.8 (0.3–23.1)	0.008
AST (IU/L), median (IQR)	240 (14–1726)	159 (14–1726)	179 (27–1540)	0.697
ALT (IU/L), median (IQR)	210 (7–931)	148 (7–931)	210 (19–769)	0.005
ALP (IU/L), median (IQR)	992 (42–4244)	590 (42–3422)	1357 (120–4244)	< 0.001
γGTP (IU/L), median (IQR)	529 (8–3445)	298 (8–3445)	737 (17–2,420)	< 0.001
LDH (IU/L), median (IQR)	332 (24–1,340)	298 (24–1340)	250 (149–1311)	0.100
T-bil (mg/dL), median (IQR)	3.8 (0.3–21.6)	2.0 (0.3–11.7)	4.7 (0.6–21.6)	< 0.001
Amylase (IU/L), median (IQR)	204 (9.2–3885)	80 (21–3885)	63 (9.2–606)	0.054
CRP (mg/dL), median (IQR)	4.0 (0.1–30)	2.1 (0.1–30)	0.9 (0.1–17)	0.074
Biliary infection (%)	129 (62.6)	98 (69.0)	31 (48.4)	0.206

*ALP* alkaline phosphatase, *ALT* alanine aminotransferase, *AST* aspartate aminotransferase, *CBD* common bile duct, *CRP* C-reactive protein, *IHBD* intrahepatic bile duct, *IQR* inter-quartile range, *LDH* lactate dehydrogenase, *γGTP* γ-glutamyl transpeptidase, *SD* standard deviation, *T-bil* total bilirubin, *WBC* white blood cells.

The final diagnoses of patients are summarized in Table 2. In total, 142 and 64 patients had a final diagnosis of benign (choledocholithiasis, 135 patients; others, 7 patients) and malignant bile duct obstruction (distal bile duct cancer, 25 patients; pancreatic cancer, 26 patients; others, 13 patients), respectively.

**Table 2:** Number of patients with benign and malignant bile duct obstruction.

Benign (%)	N = 142
Choledocholithiasis	135 (95.1)
IgG4-related sclerosing cholangitis	1 (0.7)
Chronic pancreatitis	1 (0.7)
Other benign stenoses	5 (3.5)
Malignant (%)	N = 64
Distal bile duct cancer	25 (39.0)
Pancreatic cancer	26 (40.6)
Ampullary cancer	10 (15.6)
Lymphoma	1 (1.6)
Recurrent lymph node of gastric carcinoma	1 (1.6)
Duodenal carcinoma	1 (1.6)

*IgG4* Immunoglobulin G4.

### ROC curve analysis and comparison of the predictive value of AI in differentiating malignant from benign distal bile duct obstruction

**Figure 2:**
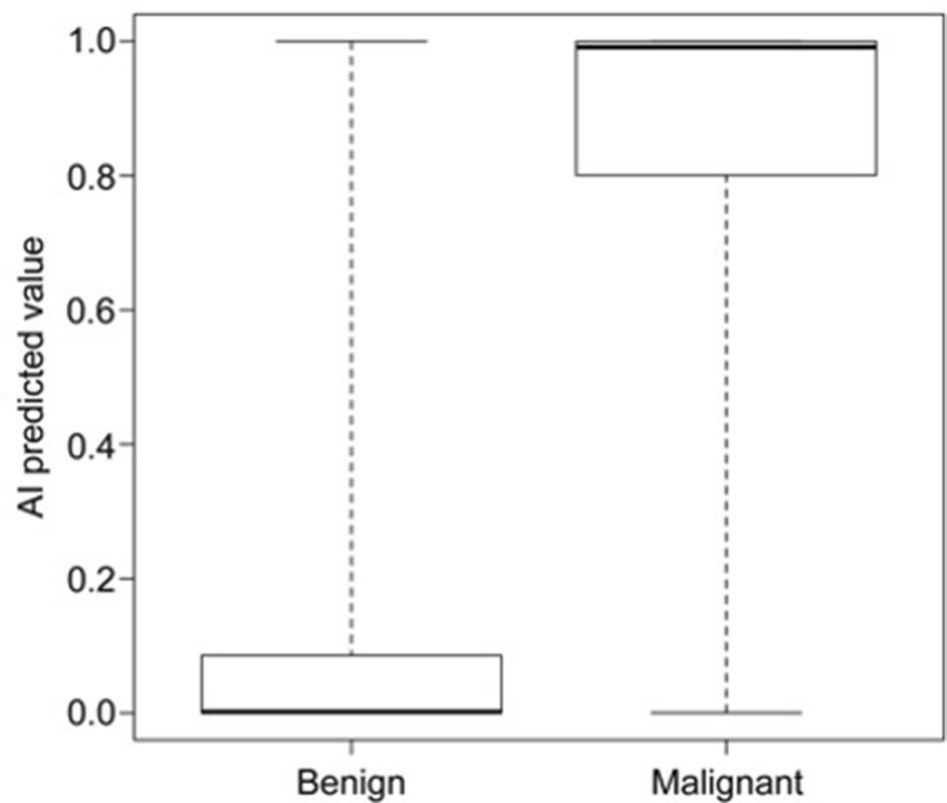
Box-and-whisker plot of the AI predictive value with benign and malignant distal bile duct obstruction. AI: artificial intelligence.

The median AI predictive value for malignant distal bile duct obstruction was significantly higher than that for benign distal bile duct obstruction (0.991 vs. 0.002, *p* < 0.001) (Fig. 2). ROC analysis was calculated to determine the cutoff value for the diagnosis of malignancy. Using an AI predictive value of 0.609 as the cutoff point resulted in the diagnostic ability of AI with a sensitivity of 83.1%, specificity of 87.2%, PPV of 74.5%, NPV of 92.0%, and accuracy of 85.9% (Table 3).

**Table 3:** Diagnostic ability of AI and other laboratory parameters in detecting malignant distal bile duct obstruction.

	Cutoff point	SE (%)	SP (%)	PPV (%)	NPV (%)	Accuracy (%)	AUROC
AI predictive value	0.609	83.1	87.2	74.5	92.0	85.9	0.908
ALP (IU/L)	749	85.9	62.4	50.5	90.7	69.4	0.795
IHBD diameter (mm)	6.1	73.4	78.6	61.8	86.3	77.0	0.794
CBD diameter (mm)	15.0	70.3	76.5	58.4	84.6	74.5	0.775

*AI* artificial intelligence, *ALP* alkaline phosphatase, *AUROC* area under the receiver operating characteristic curve, *CBD* common bile duct, *IHBD* intrahepatic bile duct, *NPV* negative predictive value, *PPV* positive predictive value, *SE* sensitivity, *SP* specificity.

**Figure 3:**
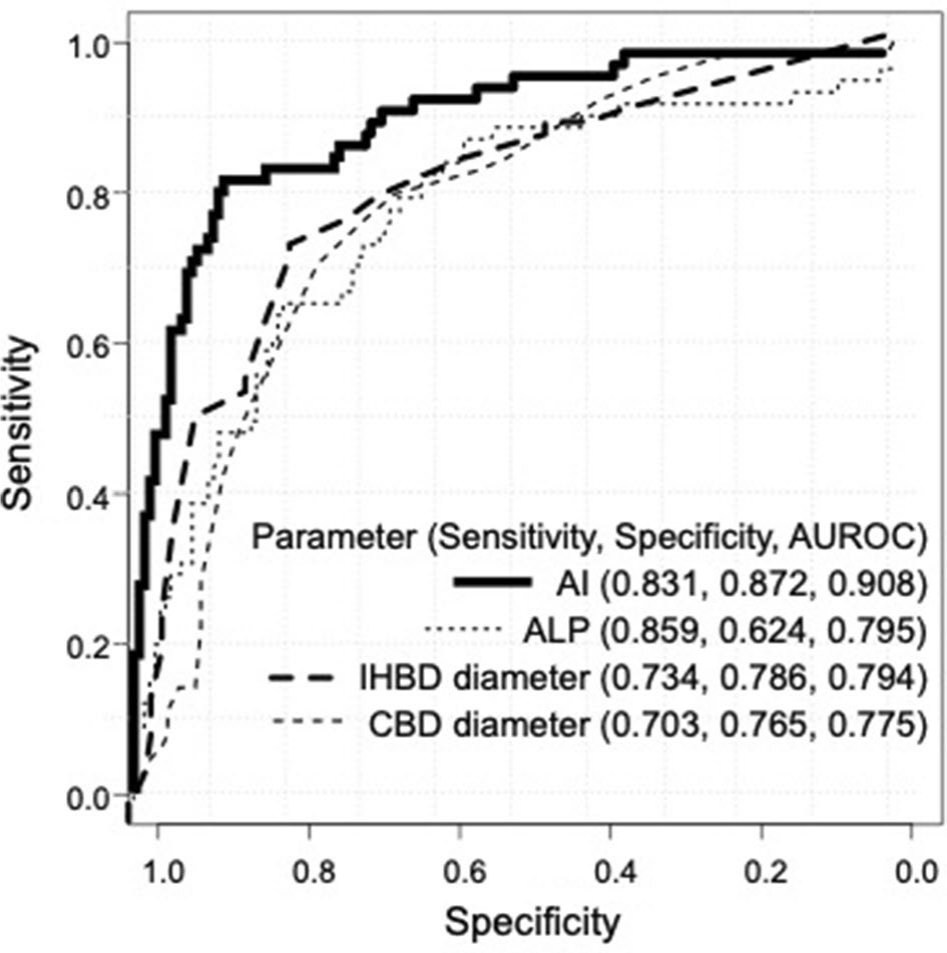
Receiver-operating characteristic (ROC) curves for artificial intelligence (AI), alkaline phosphatase (ALP), intrahepatic bile duct (IHBD) diameter, and common bile duct (CBD) diameter in differentiating malignant from benign bile duct obstruction. The areas under the ROC curve (AUROC) were as follows: AI, 0.908; ALP, 0.795; IHBD diameter, 0.794; CBD diameter, 0.775.

ROC curve analysis of the malignant predictive value of AI was demonstrated to have an AUROC of 0.908, which was significantly greater than those of ALP (0.795, *p* = 0.008), IHBD diameter (0.794, *p* = 0.007), and CBD diameter (0.775, *p* = 0.003) in diagnosing malignant distal bile duct obstruction (Fig. 3).

## Discussion

AI is a new technology for objective evaluation. The development of AI in the medical field is growing rapidly. Studies of AI tools in gastroenterology have focused primarily on endoscopy, and the number of studies evaluating these models for hepatobiliary indications using clinical parameters is few^27^. Differentiating malignant from benign bile duct obstruction is still challenging. This study is the first on the benign and malignant diagnosis of distal bile duct obstruction using AI tools with common parameters.

Our AI diagnostic model using general biomarkers showed higher diagnostic performance than blood test parameters or imaging tests. This study is unique because it shows that it may be possible to diagnose bile duct obstruction using only the general parameters performed when the patient is suspected of having bile duct obstruction. AI diagnostic models using general biomarkers might be easier to perform in a clinical setting. Biomarker-based AI may be an effective auxiliary diagnostic tool.

The development of an AI model that can accurately distinguish between benign and malignant biliary tract disorders can significantly impact a patient’s treatment strategy and prognosis. Previous reports have shown that the positive diagnosis rate of the tumor marker CA19-9 for biliary obstruction was 74%^28^. Although we could not directly compare AI and CA19-9, the AI in this study was better than that in previous reports regarding CA19-9. AI may be more useful than tumor markers in diagnosing malignancy. In addition to its diagnostic usefulness, we plan to investigate whether AI can be used as a prognostic indicator similar to tumor markers.

There are several methods for diagnosing biliary bile duct obstruction. However, contrast-enhanced CT has risks such as renal dysfunction and anaphylaxis^29^. In addition, ERCP contrast and bile duct biopsy are highly invasive, as they may lead to fatal turning points such as post-ERCP pancreatitis^30^. MRI is also useful, but there are restrictions such that it cannot be performed on patients with metallic implants^31^. This study included only general laboratory parameters that are often used clinically and can assist in making a diagnosis without using expensive machines or micro-alleys. Therefore, biomarker-based AI may be a minimally invasive and effective auxiliary diagnostic tool.

This study had several limitations. First, it was a single-center retrospective study. Second, this study had a small sample size (N = 206). Therefore, internal validation for AI diagnosis was used for malignancy (tenfold cross-validation). When assessing the diagnostic ability, it was necessary to increase the number of training and test data while separating them from each other. Aligning benign and malignant cases would decrease the number of cases. To increase the number of training data to improve the performance of AI, benign and malignant cases were not aligned. The number of benign and malignant cases differed, which may have affected the results. The apparent accuracy may have been high due to the large number of benign cases. Still, we tried to compensate for the differences as much as possible by using the synthetic minority oversampling technique and the undersampling method when creating the model for AI analysis. Third, many missing values were excluded, such as HbA1c, TG, LDL, HDL, T-chol, CEA, and CA19-9. These parameters were excluded because more than half of them had missing values. Notably, CEA and CA19-9 are important markers when determining if a lesion is benign or malignant and should have been included in this analysis. We plan to include these markers in prospective studies to further improve the diagnostic performance of benign and malignant biliary obstruction in the future. However, we propose that AI can be used as a more accurate diagnostic tool and prognostic predictor.

In conclusion, AI based on clinical biomarkers may be useful for objective evaluation prior to invasive examination and treatment of distal bile duct obstruction. This method could serve as an auxiliary for diagnosing malignant bile duct obstruction.

## Data Availability

The datasets generated and/or analyzed during the current study are available from the corresponding author on reasonable request.
